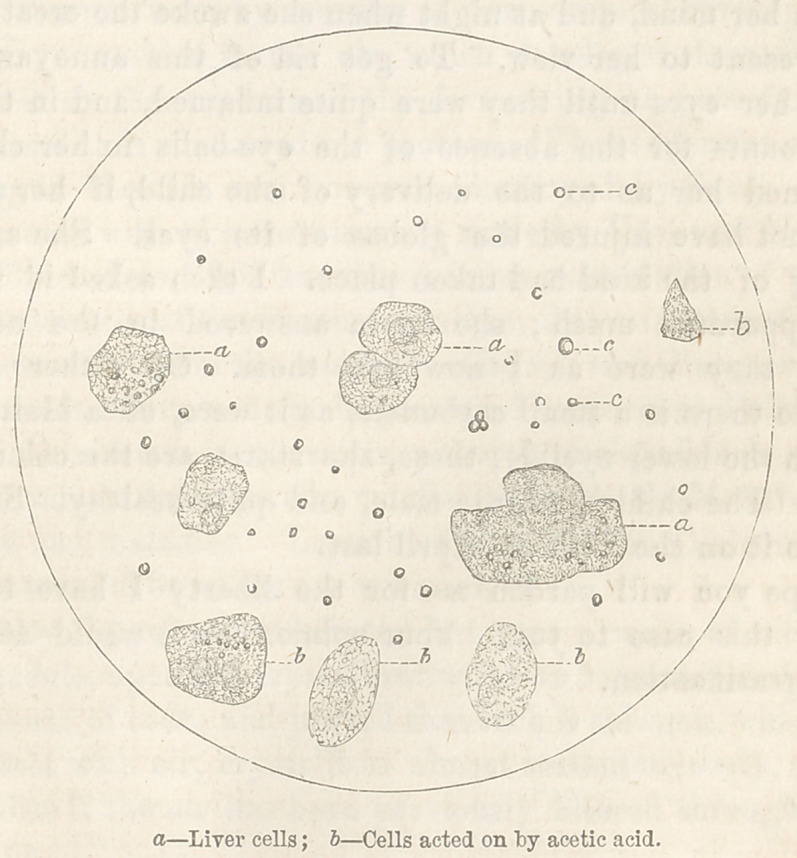# Case of Tubercular Peritonitis, with Remarks upon the Association of Oily Liver with Tubercular Phthisis

**Published:** 1854-06

**Authors:** James Darrach

**Affiliations:** Resident Physician at the Pennsylvania Hospital


### MEDICAL EXAMINER.

NEW SERIES.—NO. CXIV.—JUNE, 1854.

### ORIGINAL COMMUNICATIONS.

*Case of Tubercular Peritonitis, with remarks upon the association of oily Liver with Tubercular Phthisis.* By JAMES DARRACH, M. D., Resident Physician at the Pennsylvania Hospital.

Eliza B——, æt. eighteen years; admitted into the hospital March 17th. Previous to this time she was under the charge of Dr. F. W. Lewis, from whom I received the following account of her case before entering the hospital.

She was of healthy parents; arrived in this country from Ireland, in October, 1852, and remained well, with the exception of some irregularity in her catamenia, up to December, a year ago; she was then attacked with cough, followed by a diarrhœa of a dysenteric nature, which has continued to the present time.

In February, 1853, the abdomen began to swell, since which period her health has declined; she became emaciated, lost appetite, had hectic, and profuse night sweats.

February 10th, 1854. The abdomen much swollen; skin cool and moist; fixed pain in lumbar regions; abdominal walls thickened, so much so as to prevent any examination of the viscera beneath; superficial veins very large; auscultation and percussion of thoracic viscera, healthy; pulse 120, small and irritable; tongue red and moist; thirst; anorexia; diarrhœa; some pain with her stools, which are dark brown, slimy, and offensive; urine normal in color and quantity, contains no albumen, no sugar; sp. gr. 1015. In a subsequent examination, a few tube casts were discovered.

She was placed under the use of the liquor ferri nitratis gtt. x. thrice daily, with tinct. opii acetata gtt. x. t. d. given half an hour after the iron; was given a good diet. Under this treatment she improved; the discharges only four daily, and painless. After a short time, however, she began to sink; the fever increasing, and tongue becoming dry and red. She was then given the oil of turpentine gtt. x. thrice daily; which, disagreeing with her, was stopped, and iron, with small doses of nitrate of silver and opium, was administered.

March 17th, she entered the hospital; her condition very much as described above; she was much emaciated; abdomen swollen and tympanitic, but very tender on percussion; hectic; tongue dry, rather pale; great debility; frequent semi-fluid clay colored discharges, exceedingly offensive. Could detect no disease of thoracic viscera.

Under the impression that the girl had tubercular dyscrasia, she was placed under the use of cod liver oil f.℥ss. thrice daily, with pil. ferri carb. gr. v. t. d., and iodine ointment to be applied to the abdomen. Some little alteration took place in some of the symptoms, but no marked improvement; her anorexia remained, and the discharges from the bowels were as many as six or eight daily, very offensive. Her tongue remained dry and smooth.

Thinking that the iron was irritating the bowels, it was stopped, and the oil of turpentine prescribed, gtt. x. every three hours.

Under the use of the turpentine the motions were reduced to two or three daily, of a yellow color and less offensive, the tongue became moister, and epithelia formed along the edges. But this apparent improvement did not last long. On the 1st of April, her abdomen became more swollen, and very tender; her wrists and hands moist and cold; her mouth pinched, and her whole countenance hippocratic; the pulse became weak, frequent and thready; vomiting set in, which creosote seemed to relieve.

She died April 3d.

*Sectio Cadaveris, twelve hours after death.*

*Exterior.*—Much emaciated; abdomen swollen; no rigidity of limbs.

*Cranium.*—Not examined.

*Thorax.*—Heart healthy; about f.℥ij. of fluid in cavity of pericardium, partially washed clot in right ventricle, aorta pale interiorly, throughout its whole length. Lungs: right, contained in posterior part of middle lobe a few tubercles, which did not interfere with its crepitation; crepitant throughout; some very delicate adhesions posteriorly. Left lung: not a sign of tubercles; perfectly healthy; same delicate adhesions as in right lung, which were easily broken down; a slight deposit of tubercle in costal pleura of left side.

*Abdomen.*—Cavity contained about a gallon of yellow fluid containing pus and shreds of lymph. Peritoneum—lining of the walls contained tubercle. Liver—adherent to diaphragm above, and to the stomach and other viscera below; it was enlarged more laterally, than in its vertical diameter. The right side had its edges rounded; the left retaining much of the natural, sharp outline. The surface was of a light yellow color, pitted on pressure, and greased the knife; the centre of the lobules of a light brown color; very little blood; peritoneal covering studded with tubercle. Gall bladder empty; the extreme oily condition of this organ is shown by the drawing taken from the microscope. [*See next page.*]

Stomach: externally of a slaty hue; no tubercle on its surface; mucous membrane, through an oversight, not examined. Spleen healthy. Omentum adherent to the walls of abdomen, so that, when I cut into the cavity, I thought at first that I was cutting through the thickened parietes. It was thickened very much by the deposit of tubercle; and indeed looked

*a*—Free oil globules ; *b*—Liver cells containing oil globules.

like a mass of tubercular matter, in some places being an inch thick. Intestines, large and small were glued together, so that it was impossible to separate them; their peritoneal covering studded with tubercle, varying in size from a small pea to a grain of corn; mucous membrane of intestines was much injected; of a dark green color; the solitary glands enlarged; could discover no ulceration of Peyer's patches. Kidneys, large, softer and paler than normal. The microscope showed an excess of oil. Uterus: size normal; body of organ filled with a tubercular mass partially softened. Mesenteric glands filled with tubercle.

An interesting feature, pathologically, in the preceding case, is the occurrence of the oily liver with the deposit of tubercle. This condition of the liver in phthisis is said by some to be caused by the accumulation of hydro carbon in that organ, from its non-elimination by the diseased lungs. That this explanation is not satisfactory is shown by the observations of Rokitansky, who has remarked, that the occurrence of oily liver is quite as frequent with tubercles deposited in other parts of the body, as in the lung. How well this is proven in the foregoing case; for here we have lungs performing their full office, and yet, a more oily liver I think has seldom been seen. And going still further, if Dr. C. Handfield Jones' observations are of any weight, we may say that fatty or oily liver is not peculiar to phthisis at all.

In connection with the above remarks, I will briefly notice another case.

John E——, æt 32 years, born in Pennsylvania, of German parents, died in the Pennsylvania hospital of phthisis pulmonalis.

This subject had the right lung, or rather he may be said to have had no lung, but in its place a large cavity, having for its walls the pleura thickened with lardaceous membrane, occupying the upper part of what was the lung, while the lower half consisted of tubercle and fibrous tissue. The left lung contained much tubercle; those in the upper part had commenced softening.

*a*—Liver cells; *b*—Cells acted on by acetic acid.

Now, notwithstanding this condition of the lungs, we find the liver perfectly healthy; the amount of oil contained in the cell not exceeding that of health.

## Figures and Tables

**Figure f1:**
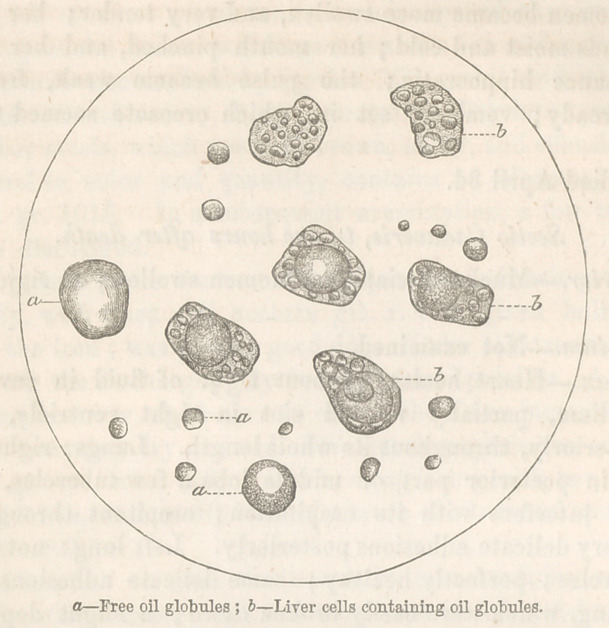


**Figure f2:**